# A Systematic Review of Comparative Efficacy of Treatments and Controls for Depression

**DOI:** 10.1371/journal.pone.0041778

**Published:** 2012-07-30

**Authors:** Arif Khan, James Faucett, Pesach Lichtenberg, Irving Kirsch, Walter A. Brown

**Affiliations:** 1 Northwest Clinical Research Center, Bellevue, Washington, United States of America; 2 Department of Psychiatry, Duke University Medical School, Durham, North Carolina, United States of America; 3 Herzog Hospital, and the School of Medicine of the Hebrew University, Department of Psychiatry, Jerusalem, Israel; 4 Program in Placebo Studies, Beth Israel Deaconess Medical Center, Harvard Medical School, Boston, Massachusetts, United States of America; 5 University of Plymouth, Plymouth, United Kingdom; 6 Department of Psychiatry and Human Behavior, Brown University, Providence, Rhode Island, United States of America; 7 Department of Psychiatry, Tufts University, Boston, Massachusetts, United States of America; University of Ulster, United Kingdom

## Abstract

**Background:**

Although previous meta-analyses have examined effects of antidepressants, psychotherapy, and alternative therapies for depression, the efficacy of these treatments alone and in combination has not been systematically compared. We hypothesized that the differences between approved depression treatments and controls would be small.

**Methods and Findings:**

The authors first reviewed data from Food and Drug Administration Summary Basis of Approval reports of 62 pivotal antidepressant trials consisting of data from 13,802 depressed patients. This was followed by a systematic review of data from 115 published trials evaluating efficacy of psychotherapies and alternative therapies for depression. The published depression trials consisted of 10,310 depressed patients. We assessed the percentage symptom reduction experienced by the patients based on treatment assignment. Overall, antidepressants led to greater symptom reduction compared to placebo among both unpublished FDA data and published trials (*F* = 38.5, df = 239, *p*<0.001). In the published trials we noted that the magnitude of symptom reduction with active depression treatments compared to controls was significantly larger when raters evaluating treatment effects were un-blinded compared to the trials with blinded raters (*F* = 2.17, df = 313, *p*<0.05). In the blinded trials, the combination of antidepressants and psychotherapy provided a slight advantage over antidepressants (*p* = 0.027) and psychotherapy (*p* = 0.022) alone. The magnitude of symptom reduction was greater with psychotherapies compared to placebo (*p* = 0.019), treatment-as-usual (*p* = 0.012) and waiting-list (*p*<0.001). Differences were not seen with psychotherapy compared to antidepressants, alternative therapies or active intervention controls.

**Conclusions:**

In conclusion, the combination of psychotherapy and antidepressants for depression may provide a slight advantage whereas antidepressants alone and psychotherapy alone are not significantly different from alternative therapies or active intervention controls. These data suggest that type of treatment offered is less important than getting depressed patients involved in an active therapeutic program. Future research should consider whether certain patient profiles might justify a specific treatment modality.

## Introduction

A number of recent articles have emphasized the inability of antidepressant medication to consistently demonstrate superiority to placebo pills [1]–[4]. Approximately half of clinical trials fail to differentiate active treatments from controls, and mean differences between drug and placebo on the Hamilton Rating Scale for Depression are small [2], [5]. This phenomenon has sparked considerable concern and criticism from the popular media, clinicians and researchers [6], [7].

Psychotherapies for depression have also come under scrutiny for their inability to demonstrate substantial superiority to various treatment controls as opposed to waiting-list (no treatment) controls [8]. Similarly, although alternative therapies such as acupuncture and exercise have shown promise in individual published studies [9], [10], the profile is less impressive according to independent reviews such as Cochrane Reviews [11], [12] and those conducted by the National Institute for Health and Clinical Excellence [13].

Given this level of ambiguity, it is unclear if pharmacological treatments are any better or worse than psychotherapies or if psychotherapies are any better than non-traditional treatments such as exercise and acupuncture. Thus, we undertook to critically evaluate relative efficacy among the various treatments for depression along with control procedures, including placebo pills.

To provide a relatively unbiased perspective of the response to treatments for depression, we used as an anchor the clinical trial data from pivotal antidepressant trials that had been submitted to the United States Food and Drug Administration (FDA) during the drug approval process. Because drug companies are required to submit information on all of the trials they conducted, these data should be free of publication and author bias [3]. Furthermore, these trials all contain data on placebo response in depression, were conducted at multiple sites and the data were accumulated over the past three decades.

Following the establishment of the anchor we conducted a literature search identifying depression clinical trials conducted over the past thirty-five years. We specifically evaluated data from depression trials that were designed to assess the role of antidepressants, psychotherapies, active intervention, and control treatments including placebo.

Our hypothesis was that the differences between the various depression treatments and controls would be relatively small. We additionally hypothesized that any differences in efficacy among these treatments would be further reduced if we applied stringent criteria for the ‘blinded’ status of the trial. This hypothesis is based on earlier reviews [8], [14]–[16] as well as our own reviews of antidepressant and placebo data from pivotal antidepressant clinical trials [2], [5].

In order to verify our hypothesis, we focused on evaluating the reduction of depressive symptoms experienced during depression trials by the patients assigned to the various active depression treatments and treatment controls. We controlled for rater bias by comparing the reduction of symptoms from depression trials with raters/clinicians who knew the study design, intent and potential treatment assignments compared to depression trials that included raters/clinicians who were blinded to these factors. As part of this exploration, we compared the antidepressant-placebo differences among unpublished, industry sponsored data obtained from the US FDA with published reports that were not sponsored by industry. Also, we evaluated if there were significant differences in the magnitude of symptom reduction among various psychotherapies.

## Methods

### Selection of Depression Trials for Evaluation

During New Drug Approval process the US FDA reviews trial level efficacy and safety data from pivotal clinical trials conducted during development programs of putative medications. In 1997 the data used by the FDA during the risk/benefit evaluations became available to the public via the Freedom of Information Act [17]. Summary Basis of Approval (SBA) reports detail data from the medication development programs and are available directly from the FDA website at www.fda.gov. If SBA reports are not available at the FDA website, the FDA staff provides data on CDRom in response to written requests.

As first part of our selection of depression treatment trials, we accessed antidepressant trial data that were reviewed by the physicians, scientists and statisticians at the FDA and reported in SBA reports. All of the data were from pivotal, placebo controlled trials that the FDA used to approve eleven antidepressants between 1987 and 2004. The efficacy dataset from these trials consisted of sixty two antidepressant clinical trials conducted between 1979 and 2001 that included 13,802 depressed patients [18], [19], [20].

Aside from this FDA data, we reviewed published literature regarding the efficacy data for traditionally accepted non-medicinal depression treatments and controls. We first searched the published literature for controlled trials of cognitive, behavioral, cognitive behavioral psychotherapies and derivatives of these treatments for major depressive disorder, dysthymia or postpartum depression. Following this search, we conducted a similar search for controlled trials of alternative therapies (exercise and acupuncture) for depression.

### Inclusion and Exclusion of Published Depression Trials

During the span of time when these depression trials were conducted there were three versions of the Diagnostic and Statistical Manual (DSM) of the APA. These consisted of DSM-II [21], DSM-III [22] and DSM-IV [23]. In essence there are no differences in the diagnostic criteria between DSM-III and DSM-IV for major depression. However, there were significant differences in the scope and definition of depression between DSM-II and DSM-III.

In order to decrease any heterogeneity, we specifically evaluated the data from trials (n = 19) that were conducted prior to the full establishment and incorporation of DSM-III that was introduced in 1978. We specifically evaluated if the precursors of DSM-III, notably Research Diagnostic Criteria (RDC) [24] or Feighner Criteria [25] were used during diagnosis. These criteria were in fact considerably narrower than DSM-III. Four of these 19 trials used Feighner criteria and five of the nineteen used RDC criteria. Among the rest (n = 10), we included the data, if specific clinical evaluations described the sample in sufficient detail to formulate DSM-III diagnostic criteria.

Although we did not follow a published pre-specified protocol during our systematic review, the trial inclusion/exclusion criteria, search stategy and primary outcome variable were defined *a-priori* (the Prisma 2009 Checklist is Figure S1). We targeted manuscripts describing depression trials of traditionally accepted and established psychotherapies or alternative therapies for Major Depressive Disorder including the depression disorder subtypes dysthymia and postpartum depression. The published trials were representative of clinically depressed ambulatory adults between the ages of 18 and 65 years of age.

We included trials that reported acute depression treatment outcomes using the Hamilton Rating Scale for Depression (HRSD), Beck Depression Inventory (BDI), or Montgomery-Asberg Depression Rating Scale (MADRS). Trials that reported an outcome in figure format were included if we were able to estimate mean total baseline and end of acute treatment outcomes from the figure.

Exclusion criteria were as follows: 1) Trials that primarily enrolled patients that were under the age of 18 or over the age of 65, 2) Trials that targeted depressed patients with major medical or psychiatric co-morbidities (e.g., human immunodeficiency virus, cancer, cardiovascular disease, patients in recovery from stroke, patients with co-morbid substance abuse), 3) Trials that did not evaluate and report treatment outcome within one week of patient of completion of treatment, 4) Trials targeting treatment resistant or hospitalized depressed patients, 5) Trials with incarcerated depressed patients, 6) Trials that were not published in the English language. Trials that were not reported in peer-reviewed journals (for example, dissertations) were also excluded.

Trials that did not report the mean baseline symptom evaluation and treatment outcome using the HRSD, MADRS or BDI were excluded. We also excluded trials that did not include an active treatment arm with a traditionally accepted psychotherapy. For example, we excluded trials that targeted experimental therapies such as bibliotherapy (telephone therapy) or computer implemented therapy without including an active treatment arm.

### Identification of Depression Trials in the Published Literature

Our primary strategy during the search for published depression treatment trials was to use a “snowball search” of the numerous published meta-analyses and reviews of psychotherapy and alternative treatments for depression.

Our literature search was conducted from September to December 2010, and targeted trials that were published between 1975 and 2009. We began by reviewing several meta-analyses designed to evaluate efficacy outcomes between psychotherapy and other treatments and controls for depression including other psychotherapies, alternative therapies, combination therapies, antidepressants, and placebos or active intervention controls [8], [16], [], [26], [27], [28,29]. During this search we identified a database of 243 psychotherapy trials compiled by Dr. Pim Cuijpers and his depression research group at www.evidencebasedpsychotherapies.org [30]. Throughout this process we retrieved title and abstract of all psychotherapy for depression trials that were used for the meta-analyses and reviews as well as those from the website by Cuijpers et al.

We then conducted a similar search targeting published controlled trials of alternative therapies for depression. We conducted this second “snowball search” by accessing Cochrane Reviews website and obtaining recently completed reviews of trials of exercise and acupuncture for treatment of major depressive disorder, dysthymia or postpartum depression [11], [12]. We retrieved title and abstract for each article that was included in the Cochrane Group evaluation of efficacy of exercise or acupuncture for depression.

After the “snowball search” of previously conducted reviews of published psychotherapy, exercise and acupuncture trials we conducted an additional online search for recently completed trials that may have been overlooked. We accessed Pubmed, Psychinfo and the Cochrane Register of Controlled Trials. We conducted identical searches in each database entering in turn the keywords acupuncture, exercise, and relaxation for trials of alternative therapies. For the psychotherapy trials we entered in turn the keywords psychotherapy, cognitive, behavioral, cognitive-behavioral, rational-emotive, and interpersonal. The terms “depression and placebo” or “depression and controlled” were used interchangeably in combination with the specific therapy names within each search engine.

For this search, we did not search for or include industry sponsored antidepressant trials that used a placebo control with an antidepressant, or that compared multiple antidepressants with no other established depression treatments, to avoid duplication of the FDA data. We only included antidepressant data from published trials of psychotherapy or alternative therapies for depression trials identified during the literature search outlined above. The antidepressant data from the published sources were independent from the pivotal registration trials that were reviewed by the FDA.

### Organization of Data

**Figure 1:**
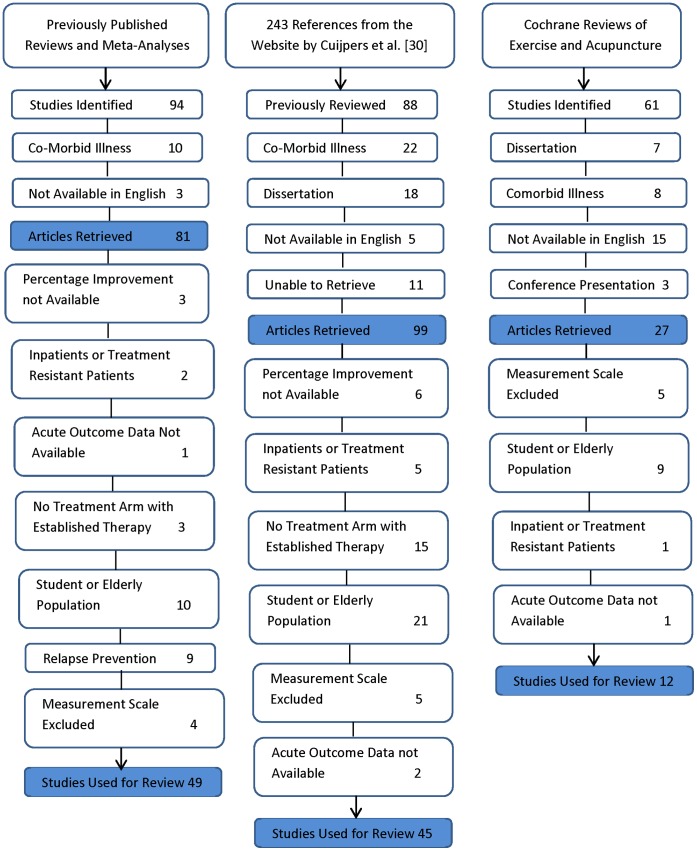
Process of Exclusion of Trials Identified During Search of Depression Treatment Reviews and Analyses, and the Website by Cuijpers and Colleagues.

The “snowball search” strategy produced 310 abstracts of depression treatment trials following our searches of previously published reviews, analyses and the website by Cuijpers et al. We reviewed the title and abstract of each article retrieved. Articles were retrieved and fully reviewed if they were available as English publications and did not specifically target depressed patients with physical or psychiatric co-morbidities such as Human Immunodeficiency Virus, cancer, cardiovascular disease, Bipolar Disorder, or Psychotic Spectrum Disorders. The PRISMA flow chart depicting process of exclusion for the published depression treatment trials is shown as Figure 1.

We included 106 trials from the search of previously published reviews and meta-analyses of published depression treatment trials. We identified an additional 9 trials with the online literature search. The list of references for the 115 depression treatment trials included in our study is shown as Appendix S1.

There were 328 treatment arms that enrolled 10,310 depressed patients in the 115 published depression treatment trials. Based on description by study authors, we identified four active treatments and four treatment controls. As shown in Table 1, the active depression treatments consisted of 218 trial arms enrolling 7,683 patients. Treatment controls consisted of 110 arms enrolling 2,627 patients.

**Table 1:** Summary Data from Depression Treatment Trials Based on Type of Treatment and Source of Data.

Treatment Type and Source	Number of Treatment Arms	Number of Patients
*FDA Summary Basis of Approval Reports*		
Investigative Agents	80	7,014
Active Comparators	31	2,220
Placebo Controls	57	4,568
Column Totals	168	13,802
*Published Depression Treatment Trials*		
***Active Treatments***		
Combination Therapy + Antidepressants	32	1,249
Antidepressants + Clinical Management	40	1,958
Accepted Psychotherapies for Depression	128	4,034
Alternative Therapies for Depression	18	442
Column Totals	218	7,683
***Treatment Controls***		
Pill Placebo Controls	16	412
Intervention Controls	48	1,095
Treatment as Usual Controls	12	530
Waiting-list Controls	34	590
Column Totals	110	2,627

Specific names of treatment arms for accepted depression treatments and treatment controls are shown as Appendix B.

The active treatments and controls, as described by the authors, are shown as Appendix S2. The active treatments were: 1) antidepressants in combination with psychotherapies or alternative treatments, 2) antidepressants with minimal clinical management, 3) psychotherapies alone that are considered to be accepted depression treatments (cognitive-behavioral, cognitive and behavioral therapies and author described derivatives), and 4) a group of treatments traditionally accepted as alternative therapies for depression. The specific names of active treatments are shown as Appendix S2 Parts1–4.

The control treatments were: 1) placebo pills, 2) procedures designated by trial authors as active intervention controls (e.g., sham acupuncture, therapies not specific to depression, partial presentations of full therapy regimens), 3) treatment-as-usual, which consisted of care by the primary care physicians or referrals to general practitioners and which may have included prescriptions for antidepressants, and 4) waiting-list controls. The specific names of the treatment controls are shown as Appendix S2 Parts 5–8.

### Designation of Blinded Status

As has been shown by several groups of researchers, the outcomes of depression trials are significantly influenced by design factors that shape the expectations of clinicians and depressed patients [31]–[33]. Other investigators [8], [14] have attempted to quantify such a possibility by evaluating the evaluator. Specifically, these earlier investigators evaluated outcome of depression trials based on the level of control that was built into trial design by blinding the symptom evaluator.

To evaluate any impact of blinding on trial outcome we used the following procedures to quantify trials as blinded or un-blinded. First, we categorized as un-blinded data from all of the depression trials that used patient ratings (BDI scale scores) as the primary dependent measure. We based this decision on the findings of Prioleau et al. that the largest treatment effects of psychotherapy relative to placebo came from studies that used undisguised self-report [14].

Second, for all trials that reported mean change in HRSD or MADRS scores we categorically evaluated the clinician/raters at the end of treatment evaluation as blinded or un-blinded following the methods outlined by Cuijpers et al. [8]. We categorized as blinded the trials that specifically described assessors at the end of treatment as independent from and blinded to the condition to which depressed patients were assigned. The trials with HRSD and MADRS that did not specify an independent symptom assessor were categorized un-blinded.

The depression trials that assigned patients to pill placebo were all categorized as blinded. Each of these trials specified that symptom assessor at end of treatment was blinded to patient assignment to antidepressant or placebo. Based on this type of demarcation, we subdivided the 115 depression treatment trials into two groups; termed group 1 with un-blinded raters (k = 59) and group 2 with blinded raters (k = 56).

### Analysis of Data

Our analysis of data was designed to evaluate the relative efficacy of the active depression treatments and controls and to evaluate the impact that blinding the trial has on treatment outcomes. We chose the mean percentage symptom reduction as our primary outcome measure. Our selection of this outcome was based on the data available as several of the published depression trials included multiple treatment arms with a single control arm violating assumptions necessary to calculate an independent effect size. There were also trials that simply did not report data from which an effect size could be calculated.

In the event that a trial reported more than one outcome measure (for example, some trials reported BDI and HRSD outcome), we selected for evaluation the clinician administered measure. Where available we recorded the Intent-to-Treat outcome, although in some cases there were not ongoing assessments throughout the trial in which case the Completer Only results were included for analysis.

**Figure 2:**
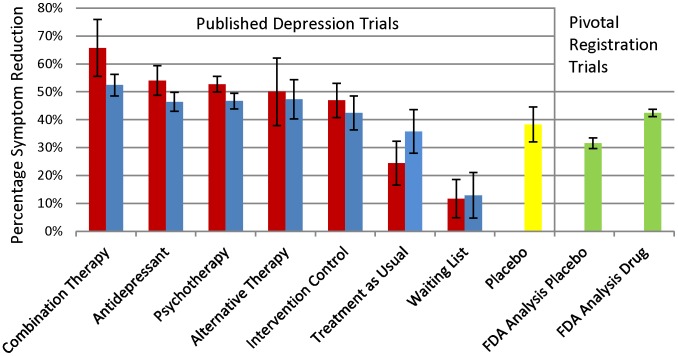
Mean Percentage Symptom Reduction from Un-blinded and Blinded Treatment Arms from Published Depression Trials Compared to Data from Pivotal Registration Depression Trials as Reported by the FDA. Red Bars Represent Un-Blinded Trial Arms Blue Bars Represent Blinded Trial Arms Yellow Represents Placebo Control Arms from Published Non-Registration trials Green Bars Represent Data from Pivotal Registration Trials The mean percentage symptom reduction was weighted by the number of assigned patients. Error Bars Represent 95% Confidence Intervals. Active treatment arms consist of combination antidepressant + therapy, antidepressants, psychotherapy, antidepressant therapy and alternative therapy. Control treatment arms consisted of placebo control, active intervention control, treatment-as-usual and waiting-list control. Blinded trials were operationally defined as those that utilized depression symptom raters that were blinded to treatment assignment of the patients.

The mean weighted percentage symptom reduction as a function of blinding, therapy type, and data source for each treatment and control is displayed in Figure 2. As a preliminary analysis, we compared the antidepressant and placebo data from published non-industry depression trials to the placebo controlled antidepressant registration trials from the FDA dataset. We conducted a 2 factor univariate analysis of variance (ANOVA). We entered as binomial independent variables the data source (1 = published data, 2 = FDA data) and the comparison of antidepressant (coded 1) versus placebo (coded 2) with percentage symptom reduction being the primary outcome measure.

This was followed by a 2×8 ANOVA of the published depression trials to evaluate the role of blinding and type of treatment on outcome for the depressed patients. To conduct this ANOVA, we entered the blinding status(1 = un-blinded, 2 = blinded) and treatment type (combination = 1, antidepressant = 2, psychotherapy = 3, alternative therapy = 4, intervention control = 5, placebo control = 6, treatment as usual = 7, waiting list = 8) as independent variables and the percentage symptom reduction was the dependent variable.

Comparative efficacy between treatment outcomes was analyzed with separate one-way ANOVAs on treatment type with blinded and un-blinded studies, respectively. Tukey’s Least Significant Difference post-hoc test was used to evaluate significance of any differences in percentage symptom reduction between the 4 active depression treatments and 4 treatment controls.

Lastly, we compared outcome of the psychotherapy treatment arms based on psychotherapy type. We conducted 2×5 ANOVA to evaluate impact of blinded status or psychotherapy type on outcome for the psychotherapy trial arms. We coded psychotherapy trial arms specifying use of Cognitive Behavioral Therapy (CBT) with 1, Cognitive Therapy with 2, and Behavioral Therapy with 3 and Interpersonal Psychotherapy with 4. Other therapies (e.g., Rational-emotive Therapy, Self-Control Therapy, Assertiveness Training, Post-Partum Support Group) were coded 5. The percentage symptom reduction was again used as dependent outcome measure weighted by number of patients. All analyses were conducted using SPSS version 19.0 (IBM).

## Results

The preliminary comparison of percentage symptom reduction with antidepressant and placebo treatment arms from published trials to antidepressant and placebo data from the FDA files revealed two significant main effects. The ANOVA indicated that antidepressants resulted in significantly greater symptom reduction than placebo, *F*(df = 239) = 38.5,*p*<.001. There was also a significant main effect of data source, *F*(df = 241) = 33.6,*p*<.001. Percentage symptom reduction was higher for published antidepressant (published data = 51%, FDA data 42 = %) and placebo (published data = 38%, FDA data = 32%) outcomes than those reported in the FDA SBA reports. The interaction between treatment type and data source was not significant, *p* = 0.313 (see Figure 2).

The 2×8 ANOVA to evaluate the role of blinding and the type of treatment on outcome in the published trials revealed a significant main effect of treatment type, *F*(df = 313) = 33.7, *p*<0.001, and a significant interaction between treatment type and blinded status, *F*(df = 313) = 2.17, p = 0.045. Treatment type was a significant predictor of percentage symptom reduction in both un-blinded and blinded trials, but the magnitude and pattern of significance differed as a function of blinding.

As shown in Figure 2, the impact of blinding was most obvious in combination therapy trials with un-blinded trials resulting in 66% percentage symptom reduction versus 53% in blinded trials. The un-blinded trials also resulted in greater symptom reduction for antidepressants, psychotherapy and intervention controls. On the other hand, treatment-as-usual resulted in 24% symptom reduction for un-blinded trials with 36% symptom reduction in blinded trials.

Results from the separate one way ANOVAs to evaluate percentage symptom reduction between treatments and controls are shown as Tables 2 and 3. As shown in Table 2, in un-blinded trials combination antidepressant + therapy resulted in greater percentage symptom reduction than psychotherapy and antidepressants alone and all of the treatment controls. There were no significant differences in percentage symptom reduction between antidepressants, psychotherapy and alternative therapy. Psychotherapy and antidepressants were superior to all of the control treatments. There was no evidence of heterogeneity of outcomes across groups of un-blinded depression treatment arms based on Levene’s Test of Equality of Error Variance, *F* = 1.82 (df = 150), *p* = 0.099.

**Table 2:** Percentage Symptom Reduction with Active Treatments and Controls among Depression Trials with an Un-blinded Rater.

	Combination	Antidepressants	Psychotherapy	Alternative Therapy	*Active Intervention Control*	*Treatment as Usual*	*Waiting List Control*
**Combination (k = 8)**	66%*						
**Antidepressants (k = 17)**	*p* = 0.044^a^	54%*					
**Psychotherapy (k = 78)**	*p* = 0.016^a^	NS^b^	53%*				
**Alternative Therapy (k = 6)**	*p* = 0.049^a^	NS^b^	NS^c^	50%*			
*Active Intervention Control (k = 22)*	*p* = 0.001^a^	*p* = 0.057^b^	*p* = 0.055^c^	NS	47%*		
*Treatment as Usual (k = 6)*	*p*<0.001^a^	*p*<0.001^b^	*p*<0.001^c^	*p*<0.001^d^	*p*<0.001^e^	24%*	
*Waiting-list Control (k = 20)*	*p*<0.001^a^	*p*<0.001^b^	*p*<0.001^c^	*p*<0.001^d^	*p*<0.001^e^	*p* = 0.016^f^	12%*

* Percentage symptom reduction values are weighted by number of patients per treatment arm for each active intervention and control for 59 un-blinded depression treatment trials with 157 treatment arms enrolling 4,083 patients.

Bolded text represents four active depression treatments. Italic text represents three treatment controls.

k = number of treatment arms for each therapy type.

Probability values show the statistical significance of comparisons between the treatment or control on the vertical access versus treatment or control on the horizontal access.

NS = Not Significant.

a Combination antidepressant therapy versus other treatments and controls.

b Antidepressant therapy versus other treatments and controls.

c Psychotherapy versus other treatments and controls.

d Alternative therapy versus treatment controls.

e Active intervention control versus treatment as usual and waiting-list controls.

f Treatment as usual versus waiting list control.

Analysis of Variance F Value (150 df) = 28.9, p<0.001, statistical significance determined with Tukey’s Post Hoc Test of Least Significant Difference.

**Table 3:** Percentage Symptom Reduction with Active Treatments and Controls among Depression Trials with a Blinded Rater.

		Combination		Antidepressants		Psychotherapy		Alternative Therapy		*Active Intervention Control*		*Placebo Control*		*Treatment as Usual*		*Waiting List Control*	
**Combination** **(k = 24)**	52%*																
**Antidepressants** **(k = 24)**	*p* = 0.027^a^		46%*														
**Psychotherapy** **(k = 50)**	*p* = 0.022^a^		NS^b^		47%*												
**Alternative Therapy (k = 12)**	NS^a^		NS^b^		NS^c^		47%*										
*Active Intervention Control (k = 26)*	*p* = 0.008^a^		NS^b^		NS^c^		NS^d^		42%*		NS^e^						
*Placebo Control* *(k = 16)*	*p*<0.001^a^		*p* = 0.030^b^		*p* = 0.019^c^		*p* = .066^d^		NS^e^		38%*						
*Treatment as Usual (k = 6)*	*p*<0.001^a^		*p* = 0.017^b^		*p* = 0.012^c^		*p* = 0.035^d^		NS^e^		NS^f^		36%*				
*Waiting-list Control* *(k = 14)*	*P*<0.001^a^		*p*<0.001^b^		*p*<0.001^c^		*p*<0.001^d^		*p*<0.001^f^		*p*<0.001^e^		*p*<0.001^g^		13%*		

* Percentage symptom reduction values are weighted by number of patients per treatment arm for each active intervention and control for 56 blinded depression treatment trials with 171 treatment arms enrolling 6,227 patients.

Bolded text represents four active depression treatments. Italic text represents the four treatment controls.

k = number of treatment arms for each therapy type.

Probability values show the statistical significance of comparisons between the treatment or control on the vertical access versus treatment or control on the horizontal access.

NS = Not Significant.

a Combination antidepressant therapy versus other treatments and controls.

b Antidepressant therapy versus other treatments and controls.

c Psychotherapy versus other treatments and controls.

d Alternative therapy versus treatment controls.

e Active intervention control versus treatment as usual and waiting-list controls.

f Placebo versus other treatment controls.

g Treatment as usual versus waiting list control.

Analysis of Variance F Value (163 df) = 11.99, p<0.001, statistical significance determined with Tukey’s Post Hoc Test of Least Significant Difference.

As shown in Table 3, in blinded trials combination therapy was superior to psychotherapy and antidepressants alone and all of the treatment controls. There were no significant differences in percentage symptom reduction between psychotherapy, antidepressants, alternative therapies, and active intervention controls. Antidepressants, psychotherapy and active intervention controls resulted in greater percentage symptom reduction than the placebo controls, treatment-as-usual and wait-list. There was no evidence of heterogeneity of outcomes across groups of blinded depression trials based on Levene’s Test of Equality of Error Variance, *F* = 1.02 (df = 163), *p* = 0.420.

**Figure 3:**
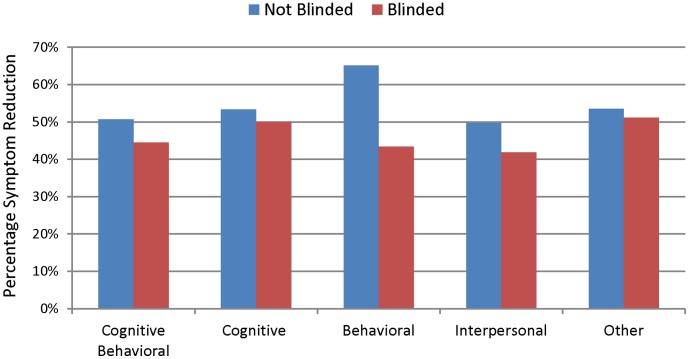
Mean Weighted Percentage Symptom Reduction of Psychotherapy Trial Arms from Published Depression Trials based on Type of Therapy Administered. The number of treatment arms for each therapy type was 24 for Cognitive Behavioral Therapy (16 un-blinded, 8 blinded), 39 for Cognitive Therapy (22 un-blinded, 17 blinded), 9 for Behavioral Therapy (7 un-blinded, 2 blinded), 14 for Interpersonal Therapy (7 un-blinded, 7 blinded) and 43 for therapies with other titles (26 un-blinded, 17 blinded).

There were no significant differences in percentage symptom reduction based on psychotherapy type as shown in Figure 3, *F*(df = 128) = 1.42, *p* = 0.23. There was a significant main effect of blinding, *F*(df = 128) = 4.11,p = 0.045.

## Discussion

Much has been made of the inability of antidepressants to demonstrate clinically significant superiority to placebo in antidepressant clinical trials. The aim of this study was to compare the efficacy of combination psychotherapy + antidepressant, antidepressants, psychotherapies, alternative therapies and controls including placebo control for depression. We also evaluated the role of blinding as a factor in assessing differences between treatments.

Not surprisingly, blinding tended to decrease improvement in active treatment arms and increase it in control arms. This finding replicates previous analyses that have evaluated treatments by quantifying the level of blinding [8], [14] suggesting that study design features do impact outcome of psychotherapy trials.

More importantly, when the raters were blinded the combined treatment of psychotherapy plus antidepressants showed only a slight advantage to antidepressants or psychotherapies alone. Although antidepressants alone and psychotherapy alone did differ significantly from placebo controls, treatment-as-usual and waiting list controls, they did not differ from alternative therapies such as exercise and acupuncture or active treatment control procedures.

It is interesting to note that although combination therapy did not statistically separate from exercise and acupuncture in the blinded trials, these alternative therapies themselves were not statistically superior to placebo (*p* = 0.066). This may be due to the small number of trials evaluating these. Aside from this fact, there is no obvious explanation for the increased variability in outcome observed in exercise and acupuncture treatment arms. Although the surface features of psychotherapy, antidepressants, exercise and acupuncture are very different, they do result in similar reduction of depressive symptoms and may have the same mechanism of action. The lack of significant differences between very diverse active treatments suggests that non-specific therapeutic factors may account for a large part of the effectiveness of these depression treatments.

Frank and Frank [34] contend that it is difficult to attribute specific outcomes to active therapies due to common therapeutic factors that patients experience during treatment. They undergo a thorough evaluation, are provided with an explanation for their distress, develop an expectation for improvement, and participate in a therapeutic ritual with an expert healer. These factors are the common threads among the conception and execution of these otherwise heterogeneous depression trials and treatments. Although such non-specific effects have been noted with comparisons of different psychotherapies for over 70 years [35], [36], our study is the first to note such outcome similarities across such a diverse group of treatments and controls for depression.

One possible reason for the lack of assay sensitivity in depression trials is that common therapeutic factors are not exclusive to active depression treatments. Although the placebo pill is in essence inert and active intervention controls are devoid of the methodological rigor of active psychotherapies, the depressed patients assigned to these conditions are exposed to all other aspects of an active therapy. This reasoning might explain the finding that patients experienced similar improvement with placebo pill as compared to those assigned to treatment as usual that may have included antidepressants.

Our study has notable limitations with respect to the inferences that can be drawn from it. We know that patients that enroll in antidepressant clinical trials are not representative of depressed patients in clinical practice [37]. Depression trials of psychotherapy, exercise and acupuncture are also likely to attract a highly select group of depressed patients [38]. Thus, the generalizability of these data is limited.

Furthermore, we were not able to evaluate the roll that severity of depression may have played on treatment outcome. We do know that a higher severity of depression contributes to increased antidepressant-placebo differences in antidepressant clinical trials [33].

These data suggest that the preference of the patient, accessibility of various treatment options and riskiness of the therapy should all be factored into depression treatment decisions. It is important to note, however, that engaging in treatment is critical to improvement. These factors should be considered during cost-effectiveness analyses of potential depression treatments. For example, allowing patients to choose a preferred treatment from outset during cost-effectiveness studies may have influence on outcome and associated cost [39], [40].

Our results also suggest that interpretation of clinical research evaluating relative efficacy of depression treatments using the randomized, double blind paradigm is problematic. With the exception of waiting-list control and treatment-as-usual, it is difficult to differentiate active treatments from “treatment controls” in adequately designed and highly blinded trials. This suggests alternative paradigms such as relapse prevention designs should be considered to evaluate potential treatments in the future.

In general, DSM depression is a broad and heterogeneous diagnosis, and researchers in the future might attempt to uncover specific profiles of depression which respond differentially to certain forms of treatment [41], [42]. Targeting treatment effects based on age, gender, weight, pattern of symptoms and biomarkers may be worth exploring.

In conclusion, our results indicate that in acute depression trials using blinded raters the combination of psychotherapy and antidepressants may provide a slight advantage whereas antidepressants alone and psychotherapy do not significantly different from alternative therapies such as exercise and acupuncture or active intervention controls such as bibliotherapy or sham acupuncture. These data suggest that type of treatment offered is less important than getting depressed patients involved in an active therapeutic program. Thus, treatment type might best be chosen on the basis of differences in the clinical presentations, risks and patient preferences and acceptance. Future research should consider whether certain patient profiles might justify a specific treatment modality.

## Supporting Information

Figure S1 Prisma 2009 Checklist.
(TIF)

Appendix S1 References and Blinded Status from 115 Depression Treatment Trials that were Included in the Analysis.
(DOCX)

Appendix S2 Active Treatment Arms (Combination Therapy + Antidepressant, Antidepressant, Psychotherapy, Alternative Therapy) and Control Treatment Arms (Placebo Control, Active Intervention Control, Treatment as Usual and Waiting-List) from 115 Published Depression Trials that Met Inclusion Criteria.
(DOCX)
