# Supplementary figures and images for: A Systematic Review of Comparative Efficacy of Treatments and Controls for Depression
Source: PLoS One. 2012 Jul 30;7(7):e41778. doi: 10.1371/journal.pone.0041778 (PMC3408478; doi:10.1371/journal.pone.0041778)

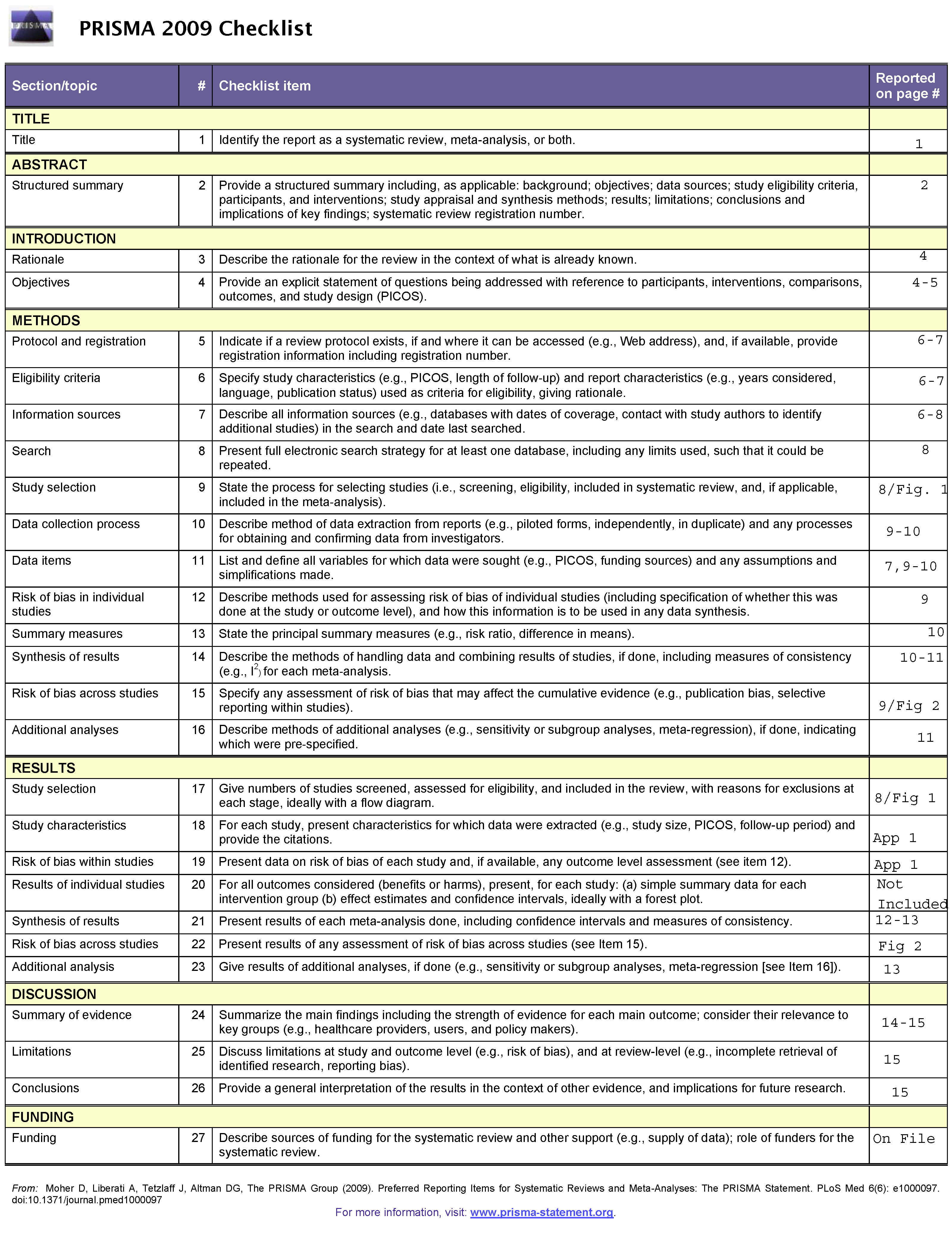

Supplement: Figure S1 — Prisma 2009 Checklist. (TIF) [file pone.0041778.s001.tif]
