# Supplementary material for: A Systematic Review of Comparative Efficacy of Treatments and Controls for Depression
Source: PLoS One. 2012 Jul 30;7(7):e41778. doi: 10.1371/journal.pone.0041778 (PMC3408478; doi:10.1371/journal.pone.0041778)
Supplement: Appendix S1 — References and Blinded Status from 115 Depression Treatment Trials that were Included in the Analysis. (DOCX) [file pone.0041778.s002.docx]

Appendix S1

Alladin A, & Alibhai A (2007) Cognitive hypnotherapy for depression: an empirical investigation. Int J Clin Exp Hypn 55: 147-166.

Allart-van Dam E, Hosman CMH, Hoogduin CAL, Schaap CPDR (2003) The coping with depression course: Short-term outcomes and mediating effects of a randomized controlled trial in the treatment of subclinical depression. Behavior Therapy 34: 381-396.

*Allen JJ, Schnyer RN, Chambers AS, Hitt SK, Moreno FA, Manber, R (2006) Acupuncture for depression: a randomized controlled trial. J Clin Psychiatry 67: 1665-1673.

*Allen JJ, Schnyer RN, Hitt SK (1998) The efficacy of acupuncture in the treatment of major depression in women. Psychol Science 9, 397-401.

*Appleby L, Warner R, Whitton A, Faragher B (1997) A controlled study of fluoxetine and cognitive-behavioural counselling in the treatment of postnatal depression. BMJ 314: 932-936.

Bagby RM, Quilty LC, Segal ZV, McBride CC, Kennedy SH et al. (2008) Personality and differential treatment response in major depression: a randomized controlled trial comparing cognitive-behavioural therapy and pharmacotherapy. Can J Psychiatry 53: 361-370.

Barnhofer T, Crane C, Hargus E, Amarasinghe M, Winder R, et al. (2009) Mindfulness-based cognitive therapy as a treatment for chronic depression: A preliminary study. Behav Res Ther 47: 366-373.

Beach SRH, O'Leary KD (1992) Treating depression in the context of marital discord: outcome and predictors of response of marital therapy versus cognitive therapy. Behavior Therapy 23: 507-528.

*Beck AT, Hollon SD, Young JE, Bedrosian RC, Budenz D (1985) Treatment of depression with cognitive therapy and amitriptyline. Arch Gen Psychiatry 42: 142-148.

Bedi N, Chilvers C, Churchill R, Dewey M, Duggan C, et al. (2000) Assessing effectiveness of treatment of depression in primary care. Partially randomised preference trial. Br J Psychiatry 177: 312-318.

*Bellack AS, Hersen M, Himmelhoch J (1981) Social skills training compared with pharmacotherapy and psychotherapy in the treatment of unipolar depression. Am J Psychiatry 138: 1562-1567.

*Bellino S, Zizza M, Rinaldi C, Bogetto F (2006) Combined treatment of major depression in patients with borderline personality disorder: a comparison with pharmacotherapy. Can J Psychiatry (Revue Canadienne de Psychiatrie) 51: 453-460.

*Beutler LE, Engle D, Mohr D, Daldrup RJ, Bergan J, et al. (1991) Predictors of differential response to cognitive, experiential, and self-directed psychotherapeutic procedures. J Consult Clin Psychol 59: 333-340.

Blackburn IM, Bishop S, Glen AI, Whalley LJ, Christie JE (1981) The efficacy of cognitive therapy in depression: a treatment trial using cognitive therapy and pharmacotherapy, each alone and in combination. Br J Psychiatry139: 181-189.

Blackburn, I. M., & Moore, R. G. (1997). Controlled acute and follow-up trial of cognitive therapy and pharmacotherapy in out-patients with recurrent depression. Br J Psychiatry 171, 328-334.

*Blumenthal JA, Babyak MA, Doraiswamy PM, Watkins L, Hoffman BM, et al. (2007) Exercise and pharmacotherapy in the treatment of major depressive disorder. Psychosom Med 69: 587-596.

*Blumenthal JA, Babyak MA, Moore KA, Craighead WE, Herman S et al. (1999) Effects of exercise training on older patients with major depression. Arch Intern Med 159: 2349-2356.

Bodenmann G, Plancherel B, Beach SR, Widmer K, Gabriel B, et al. (2008) Effects of coping-oriented couples therapy on depression: a randomized clinical trial. J Consult Clin Psychol 76: 944-954.

*Bright JI, Baker KD, Neimeyer RA (1999) Professional and paraprofessional group treatments for depression: a comparison of cognitive-behavioral and mutual support interventions. J Consult Clin Psychol 67: 491-501.

Brown RA & Lewinsohn PM (1984) A psychoeducational approach to the treatment of depression: comparison of group, individual, and minimal contact procedures. J Consult Clin Psychol 52: 774-783.

*Browne G, Steiner M, Roberts J, Gafni A, Byrne C, et al. (2002) Sertraline and/or interpersonal psychotherapy for patients with dysthymic disorder in primary care: 6-month comparison with longitudinal 2-year follow-up of effectiveness and costs. J Affect Disord 68: 317-330.

Burnand Y, Andreoli A, Kolatte E, Venturini A Rosset N (2002) Psychodynamic psychotherapy and clomipramine in the treatment of major depression. Psychiatr Serv 53: 585-590.

*Castonguay LG, Schut AJ, Aikins DE, Constantino MJ, Laurenceau J-P, et al. (2004) Integrative cognitive therapy for depression: A preliminary investigation. J Psychotherapy Integration 14: 4-20.

Clark R, Tluczek A, Brown R (2008) A mother-infant group model for postpartum depression. Infant Ment Health J 29: 514-536.

Comas-Diaz, L (1981) Effects of cognitive and behavioral group treatment on the depressive symptomatology of Puerto Rican women. J Consult Clin Psychol 49: 627-632.

Constantino MJ, Marnell ME, Haile AJ, Kanther-Sista SN, Wolman K, et al. (2008) Integrative cognitive therapy for depression: A randomized pilot comparison. Psychother Theory, Res, Practice, Training 45: 122-134.

*David D, Szentagotai A, Lupu V, Cosman D (2008). Rational emotive behavior therapy, cognitive therapy, and medication in the treatment of major depressive disorder: a randomized clinical trial, posttreatment outcomes, and six-month follow-up. J Clin Psychol 64: 728-746.

*de Jonghe F, Hendricksen M., van Aalst G, Kool S, Peen V, et al. (2004) Psychotherapy alone and combined with pharmacotherapy in the treatment of depression. Br J Psychiatry 185: 37-45.

*de Jonghe F, Kool S, van Aalst G, Dekker J, Peen J (2001) Combining psychotherapy and antidepressants in the treatment of depression. J Affect Disord 64: 217-229.

*de Mello MF, Myczcowisk LM, Menezes PR (2001) A randomized controlled trial comparing moclobemide and moclobemide plus interpersonal psychotherapy in the treatment of dysthymic disorder. Journal Psychother Pract Res 10: 117-123.

*Dekker J, Molenaar PJ, Kool S, Van Aalst G, Peen J et al. (2005) Dose-effect relations in time-limited combined psycho-pharmacological treatment for depression. Psychol Med 35: 47-58.

*DeRubeis RJ, Hollon SD, Amsterdam JD, Shelton RC, Young PR, et al. (2005) Cognitive therapy vs medications in the treatment of moderate to severe depression. Arch Gen Psychiatry 62: 409-416.

*DiMascio A, Weissman MM, Prusoff BA, Neu C, Zwilling M, et al. (1979). Differential symptom reduction by drugs and psychotherapy in acute depression. Arch Gen Psychiatry 36: 1450-1456.

*Dimidjian S, Hollon SD, Dobson KS, Schmaling KB, Kohlenberg RJ, et al. (2006) Randomized trial of behavioral activation, cognitive therapy, and antidepressant medication in the acute treatment of adults with major depression. J Consult Clin Psychol 74: 658-670.

*Doyne EJ, Ossip-Klein DJ, Bowman ED, Osborn KM, McDougall-Wilson IB, et al. (1987) Running versus weight lifting in the treatment of depression. J Consult Clin Psychol 55: 748-754.

*Dunn AL, Trivedi MH, Kampert JB, Clark CG, Chambliss HO (2005) Exercise treatment for depression: efficacy and dose response. Am J Prev Med 28: 1-8.

*Elkin I, Shea MT, Watkins JT, Imber SD, Sotsky SM, et al. (1989) National Institute of Mental Health Treatment of Depression Collaborative Research Program. General effectiveness of treatments. Arch Gen Psychiatry 46: 971-982; discussion 983.

Faramarzi M, Alipor A, Esmaelzadeh S, Kheirkhah F, Poladi K, et al. (2008) Treatment of depression and anxiety in infertile women: cognitive behavioral therapy versus fluoxetine. J Affect Disord 108: 159-164.

Fleming BM & Thornton DW (1980) Coping skills training as a component in the short-term treatment of depression. J Consult Clin Psychol 48: 652-654.

Foster RP (2007) Treating depression in vulnerable urban women: a feasibility study of clinical outcomes in community service settings. Am J Orthopsychiatry 77: 443-453.

Fremont J & Craighead LW (1987) Aerobic exercise and cognitive therapy in the treatment of dysphoric moods. Cog Ther and Res 11: 241-251.

Fuchs CZ & Rehm LP (1977) A self-control behavior therapy program for depression. J Consult Clin Psychol 45: 206-215.

Gardner P & Oei TP (1981) Depression and self-esteem: an investigation that used behavioral and cognitive approaches to the treatment of clinically depressed clients. J Clin Psychol 37: 128-135.

Grote NK, Swartz HA, Geibel SL, Zuckoff A, Houck PR (2009) A randomized controlled trial of culturally relevant, brief interpersonal psychotherapy for perinatal depression. Psychiatric Services, 60: 313-321.

*Hollon SD, DeRubeis RJ, Evans MD, Wiemer MJ, Garvey MJ et al. (1992) Cognitive therapy and pharmacotherapy for depression. Singly and in combination. Arch Gen Psychiatry 49: 774-781.

*Jacobson NS, Dobson KS, Truax PA, Addis ME, Koerner K, et al. (1996) A component analysis of cognitive-behavioral treatment for depression. J Consult Clin Psychol 64: 295-304.

*Jarrett RB, Schaffer M, McIntire D, Witt-Browder A, Kraft D, et al. (1999) Treatment of atypical depression with cognitive therapy or phenelzine: a double-blind, placebo-controlled trial. Arch Gen Psychiatry 56: 431-437.

*Keller MB, McCullough JP, Klein DN, Arnow B, Dunner DL, et al. (2000) A comparison of nefazodone, the cognitive behavioral-analysis system of psychotherapy, and their combination for the treatment of chronic depression. N Engl J Med 342: 1462-1470.

King M, Sibbald B, Ward E, Bower P, Lloyd M, et al. (2000) Randomised controlled trial of non-directive counselling, cognitive-behaviour therapy and usual general practitioner care in the management of depression as well as mixed anxiety and depression in primary care. Health Technol Assess 4: 1-83.

Krampen, G (1999) Long-term evaluation of the effectiveness of additional autogenic training in the psychotherapy of depressive disorders. Eur Psychologist 4: 11-18.

LaPointe KA & Rimm DC (1980) Cognitive, assertive, and insight oriented group therapies in the treatment of reactive depression in women. Psychother:Theory Res Practice 17: 312-321.

*Luty SE, Carter JD, McKenzie JM, Rae AM, Frampton CM, et al. (2007) Randomised controlled trial of interpersonal psychotherapy and cognitive-behavioural therapy for depression. Br J Psychiatry 190: 496-502.

Macaskill ND & Macaskill MA (1996) Rational-emotive therapy plus pharmacotherapy versus pharmacotherapy alone in the treatment of high cognitive dysfunction depression. Cog Ther Res 20: 575-592.

*Maina G, Forner F, Bogetto F (2005) Randomized controlled trial comparing brief dynamic and supportive therapy with waiting list condition in minor depressive disorders. Psychother Psychosom 74: 43-50.

*Maina G, Rosso G, Crespi C, Bogetto F (2007) Combined brief dynamic therapy and pharmacotherapy in the treatment of major depressive disorder: a pilot study. Psychother Psychosom 76: 298-305.

Malouff JM, Lanyon RI, Schutte NS (1988) Effectiveness of a Brief Group RET Treatment for Divorce-Related Dysphoria. J Rational-Emotive and Cognitive-Behavioral Therapy 6: 162-171.

*Manber R, Schnyer RN, Allen JJ, Rush AJ, Blasey CM (2004) Acupuncture: a promising treatment for depression during pregnancy. J Affect Disord 83: 89-95.

*Markowitz JC, Kocsis JH, Bleiberg KL, Christos PJ, Sacks M (2005) A comparative trial of psychotherapy and pharmacotherapy for "pure" dysthymic patients. J Affect Disord 89: 167-175.

Marshall MB, Zuroff DC, McBride C, Bagby RM (2008) Self-criticism predicts differential response to treatment for major depression. J Clin Psychol 64: 231-244.

Martin SD, Martin E, Rai SS, Richardson MA, Royall R (2001) Brain blood flow changes in depressed patients treated with interpersonal psychotherapy or venlafaxine hydrochloride: preliminary findings. Arch Gen Psychiatry 58: 641-648.

McLean PD & Hakstian AR (1979) Clinical depression: comparative efficacy of outpatient treatments. J Consult Clin Psychol 47: 818-836.

McNamara K, Horan JJ (1986) Experimental construct validity in the evaluation of cognitive and behavioral treatments for depression. J Counseling Psychol 33: 23-30.

Meager I, Milgrom J (1996) Group Treatment for Postpartum Depression: A Pilot Study. Aust N Z J Psychiatry 30: 852-860.

Milgrom J, Negri LM, Gemmill AW, McNeil M, Martin PR (2005) A randomized controlled trial of psychological interventions for postnatal depression. Br J Clin Psychol 44: 529-542.

*Miranda J, Chung JY, Green BL, Krupnick J, Siddique J, et al. (2003) Treating depression in predominantly low-income young minority women: a randomized controlled trial. JAMA 290: 57-65.

Misri S, Reebye P, Corral M, Milis L (2004) The use of paroxetine and cognitive-behavioral therapy in postpartum depression and anxiety: a randomized controlled trial. J Clin Psychiatry 65: 1236-1241.

Murphy GE, Carney RM, Knesevich MA, Wetzel RD, Whitworth P (1995) Cognitive behavior therapy, relaxation training, and tricyclic antidepressant medication in the treatment of depression. Psychol Rep 77: 403-420.

*Murphy GE, Simons AD, Wetzel RD, Lustman PJ (1984) Cognitive therapy and pharmacotherapy. Singly and together in the treatment of depression. Arch Gen Psychiatry 41: 33-41.

*Mynors-Wallis LM, Gath DH, Lloyd-Thomas AR, Tomlinson D (1995) Randomised controlled trial comparing problem solving treatment with amitriptyline and placebo for major depression in primary care. BMJ 310: 441-445.

*Neimeyer RA & Feixas G (1990) The role of homework and skill acquisition in the outcome of group cognitive therapy for depression. Behavior Therapy 21: 281-292.

Nezu AM (1986) Efficacy of a social problem-solving therapy approach for unipolar depression. J Consult Clin Psychol 54: 196-202.

*Nezu AM & Perri MG (1989) Social problem-solving therapy for unipolar depression: an initial dismantling investigation J Consult Clin Psychol 57: 408-413.

O'Hara MW, Stuart S, Gorman LL, Wenzel A (2000) Efficacy of interpersonal psychotherapy for postpartum depression. Arch Gen Psychiatry 57: 1039-1045.

Pace TM, Dixon DN (1993) Changes in depressive self-schemata and depressive symptoms following cognitive therapy. J Counseling Psychol 40: 288-294.

Pinchasov BB, Shurgaja AM, Grischin OV, Putilov AA (2000) Mood and energy regulation in seasonal and non-seasonal depression before and after midday treatment with physical exercise or bright light. Psychiatry Res 94: 29-42.

*Prendergast J, Austin M-P (2001) Early childhood nurse-delivered cognitive behavioural counselling for post-natal depression. Aust Psychiatry 9: 255-259.

*Propst LR, Ostrom R, Watkins P, Dean T, Mashburn D (1992) Comparative efficacy of religious and nonreligious cognitive-behavioral therapy for the treatment of clinical depression in religious individuals. J Consult Clin Psychol 60: 94-103.

*Quah-Smith JI, Tang WM, Russell J (2005) Laser acupuncture for mild to moderate depression in a primary care setting--a randomised controlled trial. Acupunct Med 23: 103-111.

*Ravindran AV, Anisman H, Merali Z, Charbonneau Y, Telner J, et al. (1999) Treatment of primary dysthymia with group cognitive therapy and pharmacotherapy: clinical symptoms and functional impairments. Am J Psychiatry 156: 1608-1617.

*Rehm LP, Kornblith SJ, O'Hara MW, Lamparski DM, Romano JM, et al. (1981) An evaluation of major components in a self-control therapy program for depression. Behav Mod 5: 459-489.

Revicki, DA, Siddique J, Frank L, Chung JY, Green BL, et al. (2005) Cost-effectiveness of evidence-based pharmacotherapy or cognitive behavior therapy compared with community referral for major depression in predominantly low-income minority women. Arch of Gen Psychiatry 62: 868-875.

*Ross M & Scott M (1985) An evaluation of the effectiveness of individual and group cognitive therapy in the treatment of depressed patients in an inner city health centre. J R Coll of General Practitioners 35: 239-242.

Roth D, Bielski R, Jones M, Parker W, Osborn G (1982) A comparison of self-control therapy and combined self-control therapy and antidepressant medication in the treatment of depression. Behav Ther 13: 133-144.

Rude SS (1986) Relative benefits of assertion or cognitive self-control treatment for depression as a function of proficiency in each domain. J Consult Clin Psychol 54: 390-394.

Rush AJ, Beck AT, Kovacs M, Hollon S. (1977) Comparative efficacy of cognitive therapy and pharmacotherapy in the treatment of depressed outpatients. Cog Ther and Res 1:17-31.

Rush AJ, Watkins JT (1981) Group versus individual cognitive therapy: A pilot study. Cog Ther Res 5: 95-103.

Salminen JK, Karlsson H, Hietala J, Kajander J, Aalto S, et al. (2008) Short-term psychodynamic psychotherapy and fluoxetine in major depressive disorder: a randomized comparative study. Psychother Psychosom 77: 351-357.

Sanchez VC, Lewinsohn PM, Larson DW (1980) Assertion training: effectiveness in the treatment of depression. J Clinical Psychol 36: 526-529.

Sava FA, Yates BT, Lupu V, Szentagotai A, David D (2009) Cost-effectiveness and cost-utility of cognitive therapy, rational emotive behavioral therapy, and fluoxetine (Prozac) in treating depression: a randomized clinical trial. J Clin Psychol 65: 36-52.

Schmidt MM, Miller WR (1983) Amount of therapist contact and outcome in a multidimensional depression treatment program. Acta Psychiatr Scand 67: 319-332.

*Schulberg HC, Block MR, Madonia MJ, Scott CP, Rodriguez E, et al. (1996) Treating major depression in primary care practice. Eight-month clinical outcomes. Arch Gen Psychiatry 53: 913-919.

*Scott AI & Freeman CP (1992) Edinburgh primary care depression study: treatment outcome, patient satisfaction, and cost after 16 weeks. BMJ 304: 883-887.

*Scott C, Tacchi MJ, Jones R, Scott J (1997) Acute and one-year outcome of a randomised controlled trial of brief cognitive therapy for major depressive disorder in primary care. Br J Psychiatry 171: 131-134.

Scott MJ & Stradling SG (1990) Group cognitive therapy for depression produces significant reliable change in community-based settings. Behav Psychother 18: 1-19.

Shamsaei F, Rahimi A, Zarabian MK, Sedehi M (2008) Efficacy of pharmacotherapy and cognitive therapy, alone and in combination in Major Depressive Disorder. Hong Kong J Psychiatry 18: 76-80.

Shapiro, DA, Barkham M, Rees A, Hardy GE, Reynolds S, et al. (1994) Effects of treatment duration and severity of depression on the effectiveness of cognitive-behavioral and psychodynamic-interpersonal psychotherapy. J Consult Clin Psychol 62: 522-534.

*Shaw, BF (1977) Comparison of cognitive therapy and behavior therapy in the treatment of depression. J Consult Clin Psychol 45: 543-551.

Spinelli MG & Endicott J (2003) Controlled clinical trial of interpersonal psychotherapy versus parenting education program for depressed pregnant women. Am J Psychiatry 160: 555-562.

*Strauman TJ, Vieth AZ, Merrill KA, Kolden GG, Woods TE, et al. (2006) Self-system therapy as an intervention for self-regulatory dysfunction in depression: a randomized comparison with cognitive therapy. J Consult Clin Psychol 74: 367-376.

Stravynski A, Verreault R, Gaudette G, Langlois R, Gagnier S, et al. (1994) The treatment of depression with group behavioural-cognitive therapy and imipramine. Can J Psychiatry 39: 387-390.

*Swartz HA, Frank E, Zuckoff A, Cyranowski JM, Houck PR, et al. (2008) Brief interpersonal psychotherapy for depressed mothers whose children are receiving psychiatric treatment. Am J Psychiatry 165: 1155-1162.

Taylor GT, Marshall WL (1977) Experimental analysis of a cognitive-behavioral therapy for depression. Cognitive Therapy and Research 1: 59-72.

Teri L, Lewinsohn PM (1986) Individual and group treatment of unipolar depression: comparison of treatment outcome and identification of predictors of successful treatment outcome. Behavior Therapy 17: 215-228.

^a^Veale D, Le Fevre K, Pantelis C, de Souza V, Mann A, et al. (1992) Aerobic exercise in the adjunctive treatment of depression: a randomized controlled trial. J R Soc Med 85: 541-544.

*Verduyn C, Barrowclough C, Roberts J, Tarrier T, & Harrington R (2003) Maternal depression and child behaviour problems. Randomised placebo-controlled trial of a cognitive-behavioural group intervention. Br J Psychiatry 183: 342-348.

*Watkins ER, Baeyens CB, Read R (2009) Concreteness training reduces dysphoria: proof-of-principle for repeated cognitive bias modification in depression. J abnorm psychol 118: 55-64.

Wierzbicki M, Bartlett TS (1987) The efficacy of group and individual cognitive therapy for mild depression. Cog Ther Res 11: 337-342.

*Wilson PH (1982) Combined pharmacological and behavioural treatment of depression. Behav Res Ther 20: 173-184.

*Wilson PH, Goldin JC, Charbonneau-Powis M (1983) Comparative efficacy of behavioral and cognitive treatments of depression. Cog Ther Res 7: 111-124.

Wollersheim JP, Wilson GL (1991) Group treatment of unipolar depression: A comparison of coping, supportive, bibliotherapy, and delayed treatment groups. Prof Psychol Res and Prac 22: 469-502.

Wong DF (2008) Cognitive behavioral treatment groups for people with chronic depression in Hong Kong: a randomized wait-list control design. Depress Anxiety 25: 142-148.

*Wright JH, Wright AS, Albano AM, Basco MR, Goldsmith LJ, et al. (2005) Computer-assisted cognitive therapy for depression: maintaining efficacy while reducing therapist time. Am J Psychiatry 162: 1158-1164.

*Zettle RD & Rains JC (1989) Group cognitive and contextual therapies in treatment of depression. J Clin Psychol 45: 436-445.

*Zhang WJ, Yang XB, Zhong BL (2009) Combination of acupuncture and fluoxetine for depression: a randomized, double-blind, sham-controlled trial. J Altern Complement Med 15: 837-844.

* Asterisks denote trials that were blinded

^a^ = Manuscript Reported outcome from Two Depression Treatment Trials
