# Supplementary material for: A Systematic Review of Comparative Efficacy of Treatments and Controls for Depression
Source: PLoS One. 2012 Jul 30;7(7):e41778. doi: 10.1371/journal.pone.0041778 (PMC3408478; doi:10.1371/journal.pone.0041778)
Supplement: Appendix S2 — Active Treatment Arms (Combination Therapy + Antidepressant, Antidepressant, Psychotherapy, Alternative Therapy) and Control Treatment Arms (Placebo Control, Active Intervention Control, Treatment as Usual and Waiting-List) from 115 Published Depression Trials that Met Inclusion Criteria. (DOCX) [file pone.0041778.s003.docx]

Appendix S2

| **Appendix S2 Part 1. Combination of Therapy + Antidepressant (AD)** | | | | |
| --- | --- | --- | --- | --- |
| ***Psychotherapy + Antidepressant*** | | | ***Psychotherapy + Antidepressant*** | ***Alternative Therapy + Antidepressant*** |
| Brief supportive psychotherapy + AD (1) | | | Short psychodynamic supportive psychotherapy + AD (1) | Aerobic exercise + sertraline (1) |
| Cognitive therapy + AD (8) | | | Short psychodynamic psychotherapy + AD (1) | Brief dynamic therapy + AD (1) |
| Cognitive therapy + AD (3) | | | Short term Interpersonal psychotherapy + AD (1) | Nurse-led problem solving + AD (1) |
| Cognitive-behavioral analysis system + nefazodone (1) | | |  | One cognitive-behavioral session + tasks + AD (1) |
| Interpersonal psychotherapy + AD (3) | | |  | Relaxation therapy + AD (2) |
| Psychodynamic psychotherapy + AD (1) | | |  | Sham acupuncture + AD (1) |
| Psychotherapy + AD (1) | | |  | Task assignment + AD (1) |
| Rationale emotive therapy + AD (1) | | |  | Verum acupuncture + AD + placebo (1) |
| Self-control therapy + AD (1) | | |  | Social skills training + AD (1) |
| **Appendix S2 Part 2. Antidepressant + Clinical Management** | | | | |
| ***SSRIs*** | | | ***Tricyclics*** | ***Other*** |
| Citalopram (1) | | | Amitryptiline (7) | AD not specified (6) |
| Fluoxetine (6) | | | Clomipramine (2) | Lofempram (1) |
| Paroxetine (4) | | | Desipramine (1) | Moclobemide (1) |
| Sertraline (5) | | | Imipramine *3) | Nefazodone (1) |
| Venlafaxine (1) | | | Phenelzine (1) |  |
| **Appendix S2 Part 3. Psychotherapies** | | | | |
| ***Cognitive Therapies*** | | | ***Behavioral Therapies*** | ***Cognitive-Behavioral Therapies*** |
| Cognitive therapy (39) | | | Behavioral therapy (9) | Cognitive-behavioral therapy (24) |
| Automatic thoughts training (1) | | | Assertion training (3) | Cognitive-behavioral therapy + hypnotherapy (1) |
| Brief dynamic therapy (2) | | | Behavioral activation therapy (2) | Cognitive self-control |
| Coping with depression course (2) | | | Comprehensive distancing (1) | Mother infant group therapy (1) |
| Concreteness training (1) | | | Self-control therapy (3) | Postpartum depression group (1) |
| Focused expressive therapy (1) | | | Self-system theory (1) | Psychotherapy (1) |
| Integrative cognitive therapy (2) | | |  | Rationale-emotive therapy (3) |
| Interpersonal psychotherapy (10) | | |  | Religious cognitive therapy (1) |
| Mindfulness based cognitive therapy (1) | | |  |  |
| Problem solving therapy (5) | | |  |  |
| Psychodynamic interpersonal psychotherapy (3) | | |  |  |
| Short psychodynamic supportive Psychotherapy (2) | | |  |  |
| **Appendix S2 Part 4. Alternative Therapies** | | | | |
| ***Exercise*** | | | ***Acupuncture*** |  |
| Aerobic training (3) | | | Acupuncture (3) |  |
| Autogenic training (1) | | | Laser acupuncture (1) |  |
| Exercise (8) | | |  |  |
| Running (2) | | |  |  |
|  | | |  |  |
| **Appendix S2 Part 5. Pill Placebo** | | | | |
| ***Placebo + Clinical Management (8)*** | | ***Placebo + Psychotherapy*** | | ***Placebo + Intervention Control*** |
|  | | Cognitive therapy + placebo (2) | | Placebo + 1 cognitive-behavioral therapy session (1) |
|  | | Placebo + 6 cognitive-behavioral therapy sessions (1) | | Relaxation training + placebo (1) |
|  | | Psychotherapy + placebo (1) | | Task assignment + placebo (1) |
|  | | Social skills training + placebo (1) | |  |
| **Appendix S2 Part 6. Active Intervention Controls** | | | | |
| ***Active Intervention Controls*** | ***Individual Supportive Treatment*** | | | ***Paraprofessional Therapist and Minimal Contact Treatments*** |
| Brief cognitive therapy (1) | Acupuncture control (3) | | | Bibliotherapy (2) |
| Brief supportive therapy (4) | Bright light therapy (1) | | | Computer assisted therapy (1) |
| Bogus concreteness training (1) | Flexibility training (1) | | | Counselling (4) |
| Client centered therapy (1) | Inactive laser acupuncture (1) | | |  |
| Couples coping group (1) | Low intensity stretching (1) | | |  |
| Insight oriented therapy (1) | Massage therapy (1) | | |  |
| Marital therapy (1) | Relaxation training (2) | | |  |
| Mother and toddler group (1) | Weight lifting (1) | | |  |
| Mutual support group (2) |  | | |  |
| Nondirective therapy (1) |  | | |  |
| Parenting education (1) |  | | |  |
| Partial self-control therapy (3) |  | | |  |
| Problem focused therapy (1) |  | | |  |
| **Appendix S2 Part 7. Treatment-As-Usual** | | | | |
| Community health center referral (3) |  | | |  |
| Routine primary care (6) |  | | |  |
| Treatment-as-usual (3) |  | | |  |
| **Appendix S2 Part 8. Waiting-list Control Trial Arms (34)** |  | | |  |
